# Changes in the Montagu’s Harrier Circus pygargus diet in Eastern Poland across decades promote insects and reptilians, but not birds and rodents

**DOI:** 10.1002/ece3.7416

**Published:** 2021-03-29

**Authors:** Ignacy Kitowski, Dariusz Jakubas, Paweł Mirski, Grzegorz Pitucha, Kornelia Markowska

**Affiliations:** ^1^ State School of Higher Education in Chełm Chełm Poland; ^2^ Department of Vertebrate Ecology and Zoology Faculty of Biology University of Gdańsk Gdańsk Poland; ^3^ Faculty of Biology University of Białystok Białystok Poland; ^4^ Institute of Agricultural Sciences, Land Management and Environmental Protection University of Rzeszów Rzeszów Poland; ^5^ Siemiatycze Poland

**Keywords:** changes in diet, climate change, Montagu's Harrier, pellet analysis

## Abstract

We investigated temporal changes in diet composition of the Montagu's Harrier *Circus pygargus* breeding in natural habitat (calcareous peat bog) in SE Poland. We characterized diet composition in a three‐year period (2007–2009), based on pellet analyses. We investigated whether diet composition was affected by years or stage of breeding. We compared diet of the studied population between 2000s and 1990s and with other populations. We found that the food of the studied population was dominated by insects and mammals (by number) and mammals and birds (by biomass). Biomass and abundance of main prey items differed between studied years because of different air temperatures. We found some interannual differences in contribution of some prey items including higher number of thermophilic prey (insects and amphibians) in warmer years. Comparison of pellet composition in the 1990s and 2000s revealed significant increase in the abundance of thermophilic prey (insects and reptiles) and decrease of mammals including *Microtus* voles and birds. Those changes may be linked to habitat changes in areas neighboring peat bogs and climate change‐induced changes in prey communities. The studied population was able to respond to changes in foraging habitats and prey composition by opportunistic foraging on easily available prey. The diet of the studied population is the most similar to the geographically closest populations foraging in similar habitats and characterized by high contribution of insects.

## Graphical abstract

Comparison of pellet composition of Montagu's Harrier in the 2000s revealed inter‐annual differences in abundance (a) and biomass (b) of prey items. Samples collected in the warmest 2007 [characterized by a higher contribution of of thermophilic prey (insects and amphibians)] clustered in different positions than those collected in 2008 and 2009.

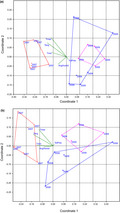

## 1 INTRODUCTION

Food availability, determined by both abundance and accessibility of food resources, is a main natural limiting factor affecting avian annual cycles (Newton, 1986, 1998). In the case of predators that actively search and hunt agile prey, additional factors such as habitat or weather may reduce accessibility through modifying prey behavior or capture probability (Schlaich et al., 2015). This may lead to food limitation despite high prey abundance (Newton, 1998; Robinson et al., 2016). Due to their top position in food webs, prey abundance is often a limiting factor for breeding density and reproductive performance, and thus, raptors are used as indicators of ecosystem status (Francksen et al., 2017; Kettel et al., 2018; Rodriguez et al., 2020; Sodhi & Ehrlich, 2010). Raptors hunt for their prey either opportunistically, that is, in proportion to their relative abundances, or because of their relative profitability (Tores et al., 2005; Seaton et al., 2008; McKinnon et al., 2013; Rodriguez et al., 2020). Studies on temporal and spatial variation in the diet composition of raptors are important to understand their foraging ecology, breeding performance, population density, or habitat selection (Litvaitis, 2000; Lopez‐Lopez et al., 2009; Di Vittorio et al., 2017).

**FIGURE 1:**
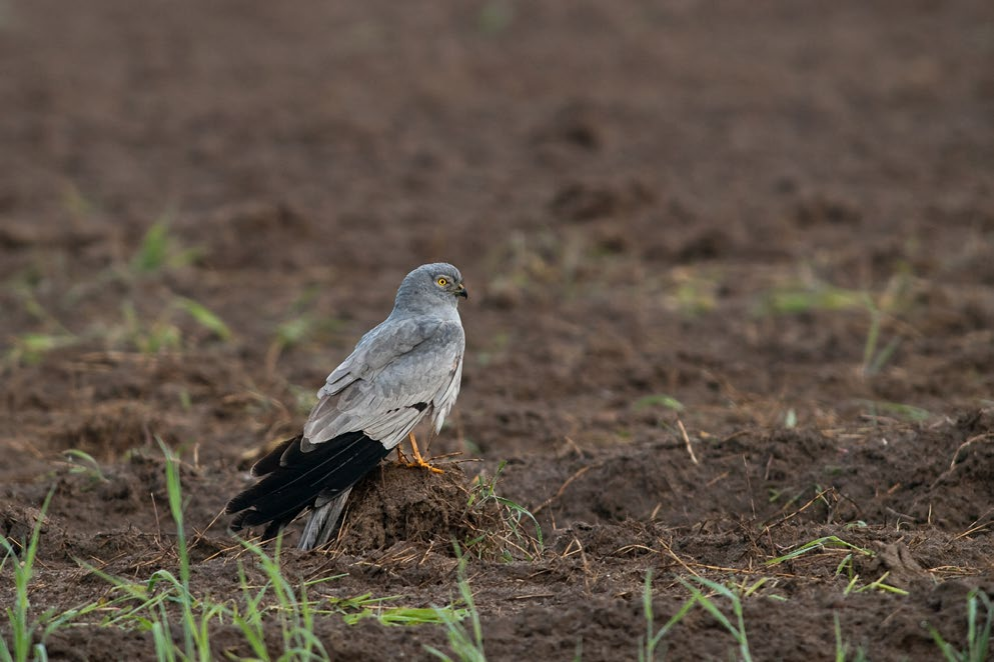
An adult male of the Montagu's Harrier *Circus pygargus*. Photograph: Andrzej Łukijańczuk

The Montagu's Harrier *Circus pygargus* is one of the smallest birds of genus *Circus* sp. (Leroux & Bretagnolle, 1996) (Figure 1). It is a migratory species breeding from North Africa, and West and Central Europe across to India and wintering in Africa and India (Clarke, 1996; Cramp & Simmons, 1980). The Montagu's Harrier is a semicolonial species, at least in some regions, with a flexible pattern of nest distribution, ranging from solitary to colonial (up to 12.7 pairs/10 ha area) (Krogulec & Leroux, 1994). It is a ground‐nester traditionally breeding in open landscapes with dense and tall herbaceous plant communities including marshes, lowland heath, rough grasslands, and steppes (Clarke, 1996; Cramp & Simmons, 1980; Terraube et al., 2010). However, in the western Palaearctic, Montagu's Harrier has started to breed in agricultural areas in recent decades, breeding in crops, mainly winter cereal or alfalfa. In the late 20th century, more than 90% of harriers in Iberia, 70%–80% in France, and 40%–50% in other western European countries nested within cereal crops (Arroyo et al., 2003). In the eastern part of Europe, increasing frequency of breeding in crops and decreasing importance of wetlands for nesting have been observed since late 1980s. Before, Montagu's Harriers were nesting entirely in wetlands in this region (Krogulec & Leroux, 1994). In Poland, until the late 1970s, nesting of the Montagu's Harrier was reported exclusively in peat bogs (Tomiałojć & Stawarczyk, 2003). Currently, up to 80%, the nests of this species are recorded in crops (Krupinski, 2017; Mirski et al., 2009).

It is an opportunistic predator preying on various birds and mammals, amphibians, reptiles, invertebrates, and avian eggs (Arroyo, 1997; Terraube & Arroyo, 2011). Diet of populations breeding in higher latitudes is dominated by mammals, especially rodents like voles (*Microtus* sp.), while southern populations are more focused on birds, reptiles, and insects (Terraube & Arroyo, 2011). Avian prey seems to serve as a very important diet component across the Montagu's Harrier distribution range. In steppe habitats, reptiles contribute considerably to total prey biomass (Terraube et al., 2010). The relative importance of particular prey items in the diet is significantly explained by their local abundance and foraging habitat characteristics, confirming the opportunistic foraging strategy of this raptor (Terraube & Arroyo, 2011). Raptors may display prey switching strategy, that is, increasing the frequency of hunting on a particular prey species when that species is abundant and decreasing when it is scarce (Murdoch, 1969). This is particularly evident for prey species that undergo seasonal or interannual massive abundance changes (Robinson et al., 2019; Steenhof & Kochert, 1988).

The aim of this study was to investigate temporal (interannual and within‐year) variation in diet (based on composition of regurgitated pellets) of Montagu's Harriers breeding in natural habitat (calcareous peat bogs) in SE Poland. We expected higher contribution of ectothermic prey (insects and reptiles) in phases of breeding season with higher air temperatures (i.e., chick‐rearing and postfledging periods) compared to the earlier phases (prelaying and incubation). We also expect higher abundance of avian prey in the diet as fledging birds are more available late in the season (Arroyo, 1997). Considering possible interannual variability in meteorological conditions, we also expected some interannual differences in pellet composition, for example, a higher frequency of ectothermic prey in warmer years. To investigate whether climatic conditions affected the presence/absence of ectothermic prey in the diet at consecutive stages of breeding, we also analyzed meteorological data in particular phases of breeding. Finally, we also compared diet composition in the studied population with historical data from the same area (1985–1995, i.e., a decade before) and with other breeding populations. We expected that due to climate and habitat changes, thermophilic prey (like insects and reptiles) will have a higher contribution to the current diet than in the past. Regarding comparison with other breeding populations, we expected that geographically closest populations (in Poland and Belarus) will be characterized by the similar diet composition.

## 2 MATERIALS AND METHODS

### 2.1 Study area

This study was conducted in calcareous peat bogs near Chełm in SE Poland in the nature reserve “Bagno Sereberyjskie” (area of 377 ha) where 7–12 pairs of Montagu's Harriers were breeding in the studied period (2007–2009). The studied reserve is situated in the center of calcareous peat bogs and is a part (47.1%) of “Błota Serebryskie” peat bog complex (area 800 ha) protected as Special Protection Area Natura 2000 PLB 060,002 (4,309 ha). The calcareous peat bog was formed as a result of accumulation of organic and mineral matter in karst valleys, eroded in calcareous rocks (Buczek, 2005; Wolejko et al., 2019). The sedge association *Cladietum marisci* with the Great Fen‐sedge *Cladium mariscus* is the dominant vegetation type in this type of peat bogs (Buczek, 2005; Fijalkowski, 1959). The Montagu's Harrier in the studied area builds its nest in clumps of sedges surrounded by water, or in partly paludine areas (Krogulec, 1992).

Direct observations revealed that peat bog, because of dense vegetation dominated by Great Fen‐sedge is rarely used by the studied population of Montagu's Harriers as hunting habitat (Krogulec, 1992; Wojtak & Kitowski, 2003; Kitowski, 1994). The study of foraging preferences of the local population revealed that only 19% foraging individuals were observed in dense vegetation dominated by Great Fen‐sedge (covering 33% of investigated area), while 48% and 30% individuals foraged in meadows (28% of investigated area) and pastures (15% of area), respectively (Kitowski, 2007). Thus, birds from local population foraged mainly in meadows, pastures, and arable land neighboring the peat bog. Those areas are characterized by a mosaic of various microhabitats including small waterbodies, groups of trees, and bushes along drainage ditches in meadows (Wojtak & Kitowski, 2003; Kitowski I.—unpublished data). Home ranges of Montagu's Harrier males in Eastern Poland were estimated to be 67 km^2^, on average, which means that the studied bog area itself cannot sustain the foraging needs and adjacent farmland is crucial for hunting (Krupiński et al., 2020).

For a long time, the calcareous peat bogs near Chełm have served as one of the most important breeding sites of Montagu's Harriers in Poland. In 1985–1993, their number was estimated to 32–43 pairs and nest density reached 4.3 pairs per 100 ha in some plots. During the study period (2007–2009), the population size was estimated at 31–34 pairs. In the 2010s, the number of breeding pairs decreased rapidly with only 2 nesting pairs in 2015 (Buczek & Buczek, 2017).

### 2.2 Fieldwork

We reconstructed the Montagu's Harrier diet composition based on prey remains in pellets. We collected them from spring to summer in 2007, 2008, and 2009. Pellets were collected from the railway embankment crossing the “Bagno Sereberyjskie” reserve. Rails, railway bollards, and shrubs at the edge of embankment served as resting, lookout, or plucking sites for the local Montagu's Harriers. There was no risk of confusing the species producing the pellets. The Montagu's Harrier is strongly territorial species and observed individuals chased away other species of raptors sitting on their perches. Moreover, pellets of other species visiting the area (Common Buzzard *Buteo buteo*, Marsh Harrier *Circus aeruginosus*) are larger compared to the studied species. Pellet collections were conducted each week, all pellets were collected, packed in zip paper bags and transported to the laboratory. In total, we collected 464 pellets.

### 2.3 Laboratory analyses

We disintegrated collected pellets in order to extract all the solid material, for example, bones, beaks, scales, and insect remains. We determined prey items using keys for the identification of mammals (Pucek, 1984) and birds (Brown et al. 1987; Jenni & Winkler, 1994). We identified mammals by bones and teeth, birds by bones, beaks, and feathers, and lizards by skulls, jaws, skin, and scales. We grouped insects not identified to the species level into the three most frequent taxa (Coleoptera, Hemiptera, and Tettigonideae). To avoid possible biases associated with a secondary predation, we excluded remains of small beetles from the analysis when they were found together with lizard scales or bird remains in the same pellets. As many collected pellets in the field were broken, to avoid multiplication of prey number from fragmented pellets, we pooled all pellet fragments collected during one visit to form one sample.

### 2.4 Meteorological data

To characterize climatic/meteorological conditions in particular stages of the Montagu's Harrier breeding period, we collected data from the closest meteorological station in Włodawa (40 km north of the studied area) from https://www.tutiempo.net/. We considered the following daily parameters: average temperature [^o^C], maximal temperature [^o^C], minimal temperature [^o^C], average relative humidity [%], and total precipitation [mm].

### 2.5 Statistical analyses

Based on the Montagu's Harrier breeding phenology, we considered the period 15 April–15 May as a prelaying period, 16 May–15 June as incubation, 16 June–15 July as chick‐rearing period, 16 July–15 August as postfledging period (Krogulec, 1992). In total, we performed three pellet collections during the prelaying period (1 in 2008 and 2 in 2009), six during the incubation (twice per year), five during the chick‐rearing period (2 in 2007, 2 in 2008, and 1 in 2009), and four during the postfledging period (3 in 2007 and 1 in 2008).

We analyzed abundance and biomass of prey remains found in pellets. We estimated abundance of particular prey items in all pellets collected during one sampling. We estimated biomass of particular prey item categories based on literature data. For mammals, we used both general values (Pucek, 1984; Salamolard et al., 2000) and values from local studies from Poland (Jedrzejewska & Jedrzejewski, 2001; Romanowski, 1988). For birds (Jedrzejewska & Jedrzejewski, 2001; Romanowski, 1988), reptiles (Jedrzejewska & Jedrzejewski, 2001), and amphibians **(**Jedrzejewska & Jedrzejewski, 2001), we used values from local studies. For insects, we used estimates from both general (Salamolard et al., 2000) and local (Bacia, 1997; Kitowski & Pawlega, 2010) studies (see values in Table 1).

**TABLE 1:** All taxa identified in the Montagu's Harrier pellets and their estimated biomass based on literature data (see references in text)

Identified taxa	Analyzed category	Estimated biomass [g]
*Sorex araneus*	Soricidae	8
*Sorex* sp.		
Soricidae		
*Neomys fodiens*		
*Talpa europaea*	*Talpa europaea*	95
*Arvicola terrestris*	*Arvicola terrestris*	130
*Microtus oeconomus*	*Microtus oeconomus*	26
*Microtus arvalis*	*Microtus arvalis*	19
*Microtus agrestis*	*Microtus* sp.	21
*Microtus* sp.		
*Criteus criteus*	Medium‐sized mammals	300
*Lepus* sp. (young)		
Medium‐sized mammals		
*Rattus norvegicus*		
*Apodemus agrarius*	Muridae	19
*Apodemus* sp.		
*Muridae*		
*Mus musculus*		
Rodentia	Rodents	19
Micromammalia		
*Alauda arvensis*	Birds	35.9
*Anthus* sp.		
*Perdix perdix*		
*Coturnix coturnix*		
Passeriformes		
Other bird remains		
Avian egg shell	Avian egg shell	2
Amphibians	Amphibians	15
*Lacerta* sp.	Reptiles	16
*Anguis* sp.		
Other Reptiles		
*Gryllus campestris*	*Gryllus campestris*	1
Coleoptera	Coleoptera	1
Tettigonideae	Tettigonideae	1
Other Insects	Other Insects	1
*Gryllotalpa gryllotalpa*		
Hemiptera		

Note

Analyzed category—categories used in analyses in this study (less numerous taxa pooled into categories).

In statistical analyses, we used only prey categories represented by at least 3 prey items. Due to relatively small sample size and unbalanced sample size for some breeding stages (lack of data from prelaying period in 2007 and from postfledging period in 2009), we firstly compared pellet composition between the breeding stages for all years combined and then between the years with all stages of breeding combined.

To compare the qualitative and quantitative composition of pellets among the stages of breeding and between years, we applied the following multivariate methods:

1. Nonmetric multidimensional scaling (nMDS), an indirect gradient analysis approach that produces an ordination based on a distance matrix explained by the Bray–Curtis similarity measure (Taguchi & Oono, 2005), to visualize dissimilarity of the studied years and stages of breeding; we also visualized environmental variables (meteorological data) in form of biplot; the correlation coefficients between each environmental variable and the NMDS scores are presented as vectors from the origin. The length of the vectors are arbitrarily scaled to make a readable biplot; thus, only their directions and relative lengths should be considered (Hammer et al., 2001); we considered the following guidelines for acceptable stress values: <0.05 = excellent, <0.10 = good, <0.20 = usable, >0.20 = not acceptable (Clarke, 1993).
2. Analysis of group similarities (ANOSIM), a procedure based on Bray–Curtis measure of similarity to test differences among groups (Clarke, 1993).
3. The similarity percentage breakdown (SIMPER) procedure to assess the average percentage contribution of individual factors to the dissimilarity between objects in a Bray–Curtis dissimilarity matrix (Clarke, 1993).

We presented nMDS graph and SIMPER results only when we found significant differences between compared categories (phase of breeding or year) in ANOSIM.

After multivariate analyses, we performed unimodal tests (Kruskal–Wallis and Mann–Whitney U tests) to compare particular prey items abundances and biomass and also meteorological conditions between the studied years.

To investigate within seasonal trends in meteorological conditions and presence of thermophilic prey (insects and reptiles) or prey with seasonal peaks of availability (birds), we used Spearman rank correlation. Due to relatively small sample size, we combined data from all years. We transformed consecutive phase of breeding into numeric values ordered in phenological order, that is, with values 1–2 for the prelaying period, 3–4 for the incubation, 5–6 for the chick‐rearing period, and 7–9 for the postfledging period.

To assess the similarity of the diet composition in the studied population and other populations of Montagu's Harriers, we used principal component analysis (PCA). We used this technique to reduce the number of variables to a few new ones called factors, representing groups of prey categories. To avoid potential method‐specific biases in diet compositions, we selected only studies based exclusively on pellets analyses. In total, we found and employed in analyses 9 studies (8 from Europe and one from Asia).

We performed nMDS, ANOSIM, SIMPER, and PCA analyses using PAST 3.0 software (Hammer et al., 2001). We expressed results as similarities in nMDS and ANOSIM and as dissimilarities in SIMPER. We performed unimodal tests and Spearman correlation in R software (R Core Team, 2017) in *ggpubr* package (Kassambara, 2019).

## 3 RESULTS

### 3.1 Diet composition

**FIGURE 2:**
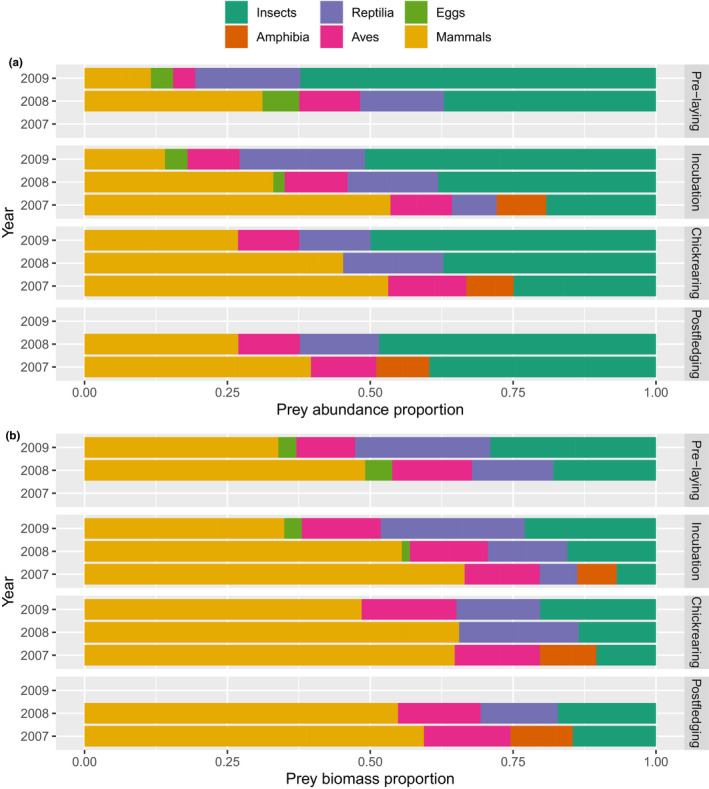
The Montagu's Harrier main prey abundance (a) and biomass (b) in pellets collected during the particular phases of the breeding season in 2007–2009 in calcareous peat bogs in SE Poland

In total, we collected 464 pellets (127 in 2007, 179 in 2008, and 158 in 2009). Mean number of prey items per pellet was the highest during postfledging period and the lowest during the chick‐rearing period (Table 2). We identified six main types of prey in the pellets (Figure 2; Tables 1 and 2). Insects were the most abundant (70.7%) prey category; however, their contribution to the biomass was very small (4.7%) (Table 2). Only during the postfledging period insects made up 6.8% of total biomass. The most numerous insect, the Field Cricket *Gryllus campestris* was mainly preyed during the prelaying period.

**TABLE 2:** Abundance and biomass of prey items found in pellets of Montagu's Harrier breeding in calcareous peat bogs near Chełm (SE Poland) during prelaying (plg), incubation (inc), chick‐rearing (chr), and postfledging (pfp) periods and in all stages of breeding combined (Tot) in 2007–2009 combined

Prey item	*N*	Prey abundance [%]					Prey biomass [%]				
		plg	inc	chr	pfp	Tot	plg	inc	chr	pfp	Tot
*Soricidae*	5	–	0.2	–	0.8	0.3	–	0.1	–	1.3	0.2
*Talpa europaea*	8	–	1.2	0.4	0.2	0.5	–	**6.7**	2.9	3.6	3.7
*Arvicola terrestris*	11	0.7	0.5	1.4	–	0.7	**6.6**	2.3	**13.9**	–	**7.0**
*Microtus oeconomus*	27	–	1.0	3.8	0.8	1.6	–	0.9	**7.6**	3.9	3.4
*Microtus arvalis*	64	1.5	**5.8**	**5.6**	1.6	3.8	1.9	**5.0**	**8.1**	**5.7**	**6.0**
*Microtus* sp.	92	4.7	**10.0**	**5.6**	2.0	**5.5**	**6.9**	**11.9**	**9.0**	**7.9**	**9.5**
*Muridae*	10	0.4	0.5	0.8	0.6	0.6	0.5	0.7	1.2	2.1	0.9
Rodents	45	3.6	3.6	2.6	1.4	2.7	4.8	3.7	3.8	**5.1**	4.2
Medium–sized mammals	26	2.5	2.2	1.4	0.6	1.5	**53.0**	**37.2**	**32.1**	**34.0**	**38.3**
Birds	71	3.6	**5.8**	**5.0**	2.4	4.2	**9.0**	**13.3**	**13.7**	**16.3**	**12.5**
Reptiles	98	**12.0**	**12.7**	1.6	1.0	**5.8**	**12.9**	**11.8**	1.9	2.9	**7.5**
Amphibians	27	–	2.9	1.4	1.6	1.6	–	3.2	1.6	4.5	2.0
Other Insects	732	2.9	**8.5**	**60.1**	**78.9**	**43.6**	0.2	0.3	3.5	**11.3**	2.8
*Gryllus campestris*	410	**62.9**	**38.2**	**9.8**	**6.3**	**24.4**	3.9	2.5	0.7	1.1	1.8
*Coleoptera*	12	0.7	1.7	0.4	0.2	0.7	0.0	0.1	0.0	0.0	0.0
*Tettigonideae*	34	2.9	4.6	–	1.4	2.0	0.1	0.2	–	0.2	0.1
Avian egg shell	6	1.5	0.5	–	–	0.4	0.2	0.1	–	–	0.1
Total	1678	100	100	100	100	100	100	100	100	100	100
No. of pellets	464	67	131	178	88						
Mean No. of prey items per pellet		4.10	3.14	2.81	5.60						

Note

Dominant prey items (with abundance or biomass > 5%) are bolded. *N*—number of prey items of particular category.

Amphibians had negligible contribution, both to the total abundance and to the biomass (1.6 and 2.0%, respectively).

Reptiles constituted 5.8% and 5.6% of total abundance and biomass, respectively. During the prelaying and incubation period, their contribution to the diet was higher, making up 10.3% and 8.0% of prey biomass, respectively. Among identified remains of reptiles, those belonging to lizards *Lacerta* sp. were the most numerous (86.7%) (Table 1).

Birds were not a very abundant prey group, making up only 4.2% of the total share. However, their contribution to the total biomass was much higher (12.5%) (Table 2). During nesting and postfledging periods, their contribution to the diet biomass was higher, making up 12.9% and 11.8%, respectively (Table 2). Gray Partridge *Perdix perdix* and Common Quail *Coturnix coturnix* were the largest prey of the studied harriers. Remains of both species were found during incubation period in 2008. Remains of avian eggs (eggshells) were found in pellets sporadically during prelaying and incubation periods.

Mammal remains made up only 17.2% of abundance but they dominated in biomass (76.3%). The most numerous group of mammalian prey, voles (*Microtus* sp., *M. oeconomus,* and *M. arvalis* combined) made up 37.8% of abundance but only 18.6% of biomass. During the chick‐rearing periods, voles made up 15.0% of abundance and 24.7% of biomass. Medium‐sized mammals [including European Hamster *Cricetus cricetus*, Norway Rat *Rattus norvegicus,* and hares *Lepus* sp. (probably carrion)] played an important role in biomass constituting from 32% of the total biomass during chick‐rearing to 53% during prelaying period (Table 2). In this group, the European Hamster was the largest mammalian prey found in this study. It was found in pellets in 2007 and 2009. Synanthropic mammals (Brown Rat and House Mice *Mus musculus*) were hunted mainly during prelaying and incubation periods, with 7%–8% contribution to the total diet biomass.

### 3.2 Within‐year variation in diet and meteorological conditions

We found significant differences between the phases of breeding (for all years combined) neither in abundance nor in biomass of all prey categories (ANOSIM with Bray–Curtis as distance measure: abundance: *R *= 0.071, *p* = 0.233, biomass: *R *= 0.0193, *p* = 0.379).

We found significant relationship neither between phase of breeding and meteorological conditions nor between phase of breeding and abundance of various prey items (for all years combined) (Table 3).

**TABLE 3:** Relationship between phase of breeding (coded as 1–2—prelaying period, 3–4—incubation, 5–6—chick‐rearing period, and 7–9—postfledging period) and meteorological conditions and abundance of various prey items (Spearman rank correlation)

Phase of breeding versus…	rho	S	*p*
Meteorological conditions			
Mean temp.	0.093	878.6	0.713
Minimal temp.	0.379	602.1	0.121
Maximal temp.	0.354	625.8	0.149
Mean humidity	0.216	759.7	0.389
Total precipitation	0.277	700.8	0.266
Prey items			
Amphibians	0.079	892.7	0.756
Birds	0.072	899.5	0.777
Reptiles	−0.226	1,188.4	0.366
Other Insects	−0.078	1,044.6	0.758
*Gryllus campestris*	−0.297	1,256.4	0.232

### 3.3 Interannual diet variation

We found significant differences in abundance and biomass of all prey categories between the studied years (for all phases of breeding combined). In the case of abundance (ANOSIM with Bray–Curtis as distance measure: *R *= 0.781, *p* = 0.0001), pairwise comparisons (with Bonferroni‐corrected values) revealed significant differences in prey abundance between 2007 and 2008 (*p* = 0.004), and 2007 and 2009 (*p* = 0.003). The values in 2008 and 2009 were similar (*p* = 0.148). Similarly in the case of biomass (ANOSIM: *R *= 0.612, *p* = 0.0002), pairwise Bonferroni‐corrected comparisons revealed significant differences in prey biomass between 2007 and 2008 (*p* = 0.004), and 2007 and 2009 (*p* = 0.005). The values in 2008 and 2009 were similar (*p* = 0.364).

**FIGURE 3:**
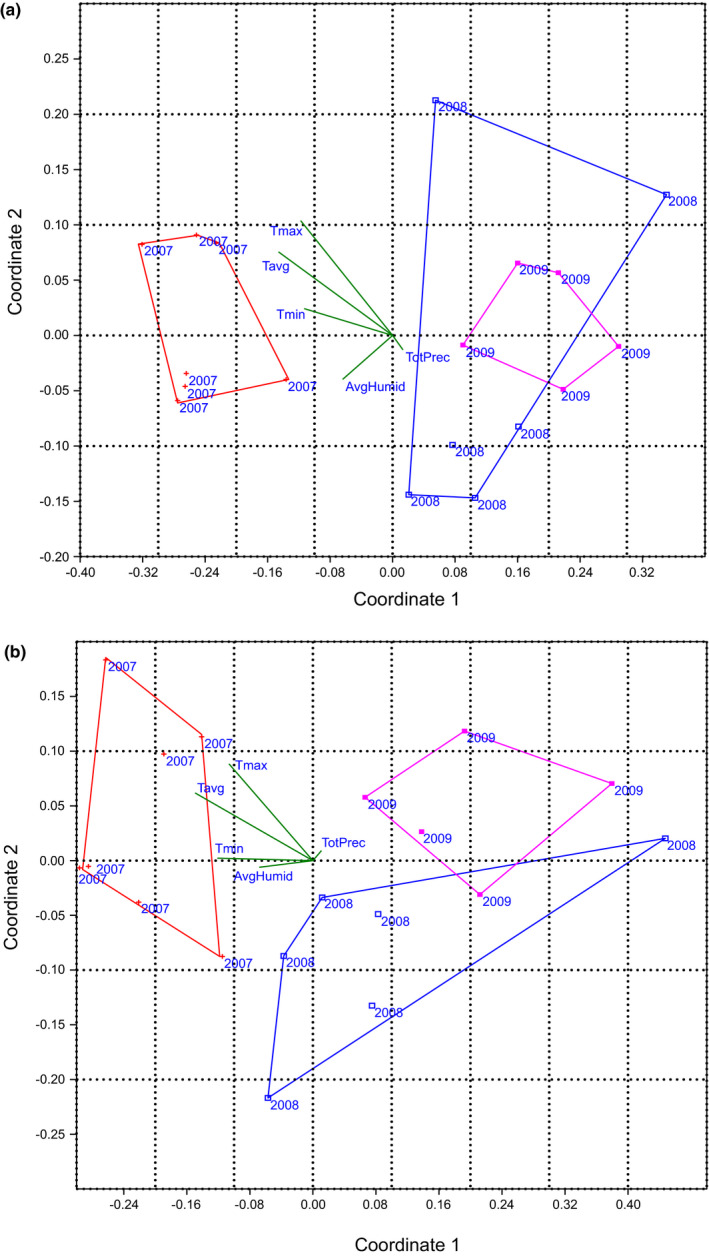
nMDS biplot of Bray–Curtis similarities in abundance (a) and biomass (b) of prey in pellets of Montagu's Harrier breeding in calcareous peat bogs near Chełm in SE Poland. Convex hulls contain all samples from one year. Meteorological data codes: AvgT—average air temperature (^o^C), TMax—maximal air temperature (^o^C), TMin—minimal air temperature (^o^C), AvgHum—average relative humidity (%), and TotPrec—total precipitation (mm)

The nMDS plot of similarity in the abundance of prey in the Montagu's Harrier pellets (with a good value of stress – 0.107) showed that samples from 2007 clustered in different positions than from 2008 and 2009. Clusters representing 2008 and 2009 strongly overlapped (Figure 2a). Also, in the case of biomass, nMDS plot (with a usable stress value ‐ 0.136) showed that pellets composition in particular years generally clustered in different positions. Only clusters representing 2008 and 2009 partly overlapped (Figure 3b).

We found that other insects and Field Cricket *Gryllus campestris* contributed the most (16.5% and 16.1%, respectively) to the pattern of overall average dissimilarity (44%) in prey items abundance between years (SIMPER) (Table 4). Medium‐sized mammals, reptiles, and *Microtus arvalis* contributed the most (9.8%, 8.9%, and 8.9%, respectively) to the pattern of overall average dissimilarity (52%) in biomass (SIMPER) (Table 4).

**TABLE 4:** Sources of variability in abundance and biomass of prey in pellets of Montagu's Harrier breeding in calcareous peat bogs near Chełm in SE Poland (log‐transformed) (average percentage dissimilarity for all years with all stages of breeding combined), according to a SIMPER analysis with Bray–Curtis similarity measure; only elements with a contribution of > 5% are presented

Overall abundance			Overall biomass		
Prey item	AD	CO	Prey item	AD	CO
Other Insects	10.0	16.5	Medium‐sized mammals	5.1	9.8
*Gryllus campestris*	9.7	16.1	Reptiles	4.6	8.9
Reptiles	5.3	8.8	*Microtus arvalis*	4.6	8.9
*Microtus arvalis*	4.8	8.0	Birds	4.0	7.8
Birds	3.8	6.3	*Microtus oeconomus*	4.0	7.6
Amphibians	3.2	5.3	*Gryllus campestris*	3.9	7.5
*Microtus* sp.	3.2	5.2	Other Insects	3.8	7.4
*Microtus oeconomus*	3.1	5.1	*Arvicola terrestris*	3.7	7.2
			Amphibians	3.5	6.7
			*Talpa europaea*	3.3	6.4
			Rodents	2.9	5.6
			Muridae	2.8	5.4

Note

AD, average dissimilarity and CO, contribution [%].

**FIGURE 4:**
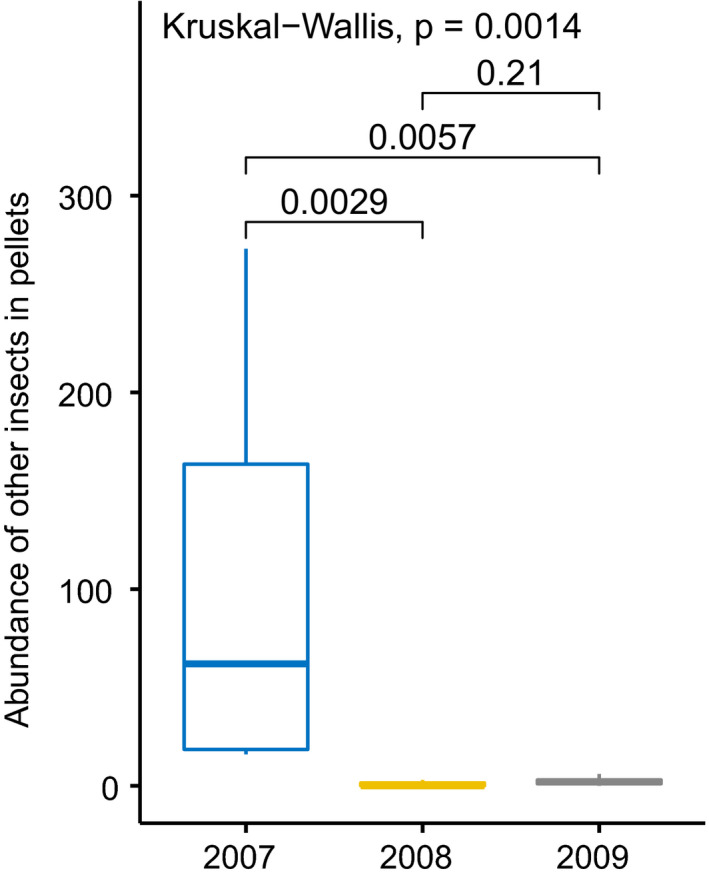
Abundance of the prey item category “other insects” characterized by the highest interannual dissimilarity in SIMPER analyses. Boxplots show the median (band inside the box), the first (25%) and third (75%) quartile (box), the lowest and the highest values within 1.5 interquartile range (whiskers) and outliers (dots). Values above lines indicate p values for post hoc test (Wilcoxon test)

Unimodal tests for prey items contributing the most to the pattern of overall average dissimilarity in abundance revealed that the number of other insects was the highest in 2007. It was significantly higher compared to 2008 and 2009. Abundance of this prey item was similar in 2008 and 2009 (Figure 4).

**FIGURE 5:**
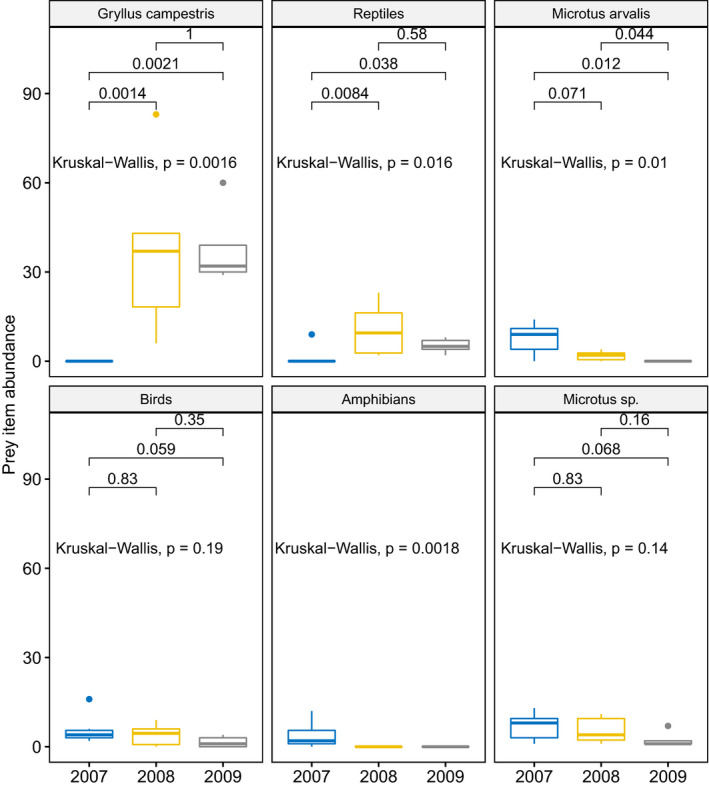
Abundance of the prey items characterized by the highest interannual dissimilarity in a SIMPER analyses. Boxplots show the median (band inside the box), the first (25%) and third (75%) quartile (box), the lowest and the highest values within 1.5 interquartile range (whiskers) and outliers (dots). Values above lines indicate p values for post hoc test (Wilcoxon test)

Among other prey categories, we found significant interannual differences in abundance of Field Crickets (with higher numbers in 2008 and 2009 compared to 2007), reptiles (with higher number in 2008 and 2009 compared to 2007), *Microtus arvalis* (with higher numbers in 2007 and 2008 compared to 2009), and amphibians (the highest value in 2007) and lack of differences for birds and *Microtus* sp. (Figure 5).

**FIGURE 6:**
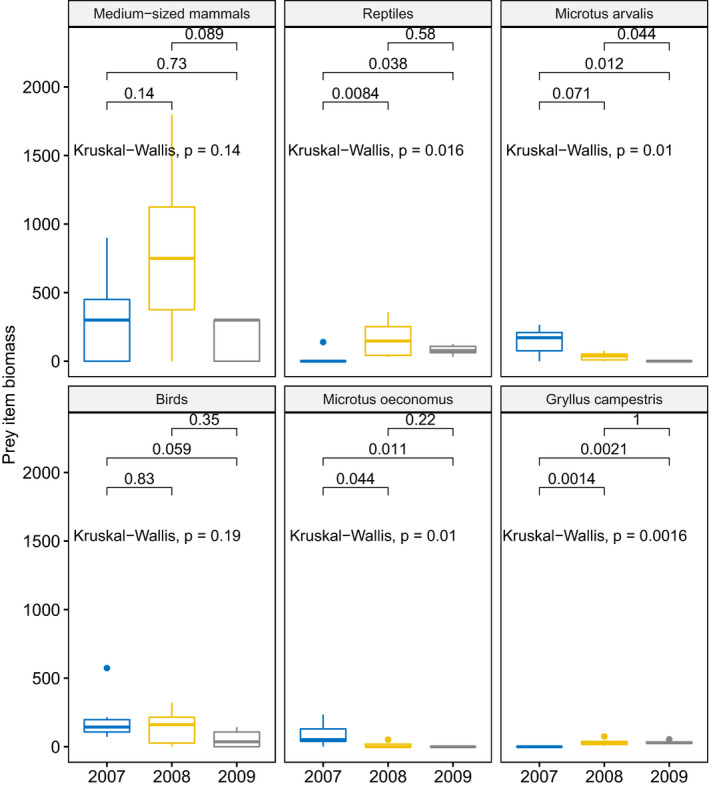
Biomass of the prey items characterized by the highest interannual dissimilarity in a SIMPER analyses. Boxplots show the median (band inside the box), the first (25%) and third (75%) quartile (box), the lowest and the highest values within 1.5 interquartile range (whiskers) and outliers (dots). Values above lines indicate p values for post hoc test (Wilcoxon test)

Unimodal tests for prey items contributing the most to the pattern of interannual overall average dissimilarity in biomass revealed interannual differences (Kruskal–Wallis test *p* < 0.02) except for medium‐sized mammals and birds (*p* > 0.13) (Figure 6). Reptiles were characterized by higher biomass in 2008 and 2009 compared to 2007, *Microtus arvalis* and *Microtus oeconomus* by higher values in 2007 and 2008 compared to 2009, and Field Cricket by higher biomass in 2008 and 2009 compared to 2007 (Figure 6).

### 3.4 Interannual variation in meteorological conditions

**FIGURE 7:**
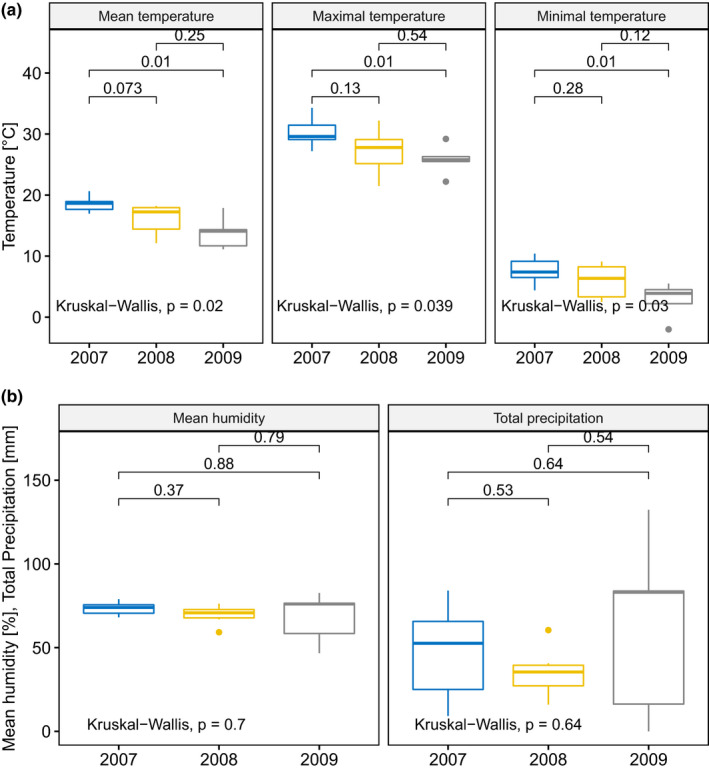
Interannual differences in daily air temperatures (a), mean humidity and total precipitation (b) recorded during the breeding period (all stages combined) of Montagu's Harriers in Włodawa meteorological post (40 km from the studied area) in the studied years. Boxplots show the median (band inside the box), the first (25%) and third (75%) quartile (box), the lowest and the highest values within 1.5 interquartile range (whiskers) and outliers (dots). Values above lines indicate p values for post hoc test (Wilcoxon test)

Unimodal tests revealed that mean, max, and min air temperatures in 2007 were significantly higher than in 2009. Differences in air temperature between 2007 and 2008, and 2008 and 2009 were not significant (Figure 7a). Mean humidity and total precipitation were similar in all studied seasons (Figure 7b).

### 3.5 Temporal variation in the Montagu's Harrier diet

Previous studies of the Montagu's Harrier diet performed in the same areas during prelaying (1992–1995) (Wiacek & Niedzwiedz, 2005), chick‐rearing (1985–1988) (Krogulec, 1992), and post fledging (1989–1992) (Kitowski, 1994) periods (Table 5) allowed us to investigate temporal changes in diet composition in last decades. Our comparison revealed some significant temporal trends at particular stages of breeding. During the prelaying period, the number of hunted birds, voles, other nonvoles mammals, and avian eggs in 1992–1995 was significantly lower than in our study (Table 5). We found the opposite pattern at this stage of breeding for reptiles and insects being most numerous in the current study. During the chick‐rearing period, Montagu's Harrier pellets in 1985–1989 contained significantly less birds, voles and no voles and more insects compared to 2007–2009 (Table 5). Those temporal trends correspond well with those observed during the preplaying period. During the postfledging period, Montagu's Harriers in 1989–1992 consumed less birds and voles, and more other mammals compared to 2007–2009.

**TABLE 5:** Abundance and relative number of particular prey categories identified in the pellets of Montagu's Harrier nesting in calcareous peat bogs near Chełm (SW Poland) in various phases of the breeding period in the 1990 and 2000s

Prey category	Prelaying period				Chick‐rearing period				Postfledging period			
	1992–1995 [1]	2007–2009 [2]	Change [1] versus [2]		1985–1989 [3]	2007–2009 [2]	Change [3] versus [2]		1999–1993 [4]	2007–2009 [2]	Change [4] versus [2]	
	*N* (%)	*N* (%)	*p*	Trend	*N* (%)	*N* (%)	*p*	Trend	*N* (%)	*N* (%)	*p*	Trend
Amphibians	3 (0.9 )	0 (0)	–	↓	0 (0)	7 (1.4)	–	↑	2 (0.9)	8 (1.6)	0.511	=
Reptiles	17 (4.9)	33 (12.0)	0.002	↑	3 (0.6)	8 (1.6)	0.132	=	0 (0)	5 (1.0)	–	↑
Birds	51 (14.8)	10 (3.6)	<0.001	↓	141 (26.2)	25 (5.0)	<0.001	↓	29 (12.3)	12 (2.4)	<0.001	↓
*Microtus* sp.	54 (15.7)	18 (6.5)	<0.001	↓	144 (26.8)	75 (15.0)	<0.001	↓	60 (25.4)	21 (4.3)	<0.001	↓
Other mammals	64 (18.6)	19 (6.9)	<0.001	↓	112 (20.8)	33 (6.6)	<0.001	↓	10 (4.2)	19 (3.9)	0.844	=
Insects	134 (39.0)	191 (69.5)	<0.001	↑	132 (24.5)	351 (70.4)	<0.001	↑	135 (57.2)	428 (86.8)	<0.001	↑
Other prey	21 (6.1)	4 (1.5)	0.003	↓	6 (1.1)	0 (0)	–	↓	0 (0)	0 (0)	–	–
Total	344 (100)	275 (100)			538 (100)	499 (100)			236 (100)	493 (100)		

Note

References: [1]—Wiącek and Niedzwiedz (2005), [2]—present study, [3]—Krogulec (1992), [4]—Kitowski (1994).

*p*, *p* value for interperiod comparison: χ^2^ or Fisher's exact test, trends: ↓, decrease, ↑, increase, =, no significant change.

### 3.6 Comparison to other breeding populations

**FIGURE 8:**
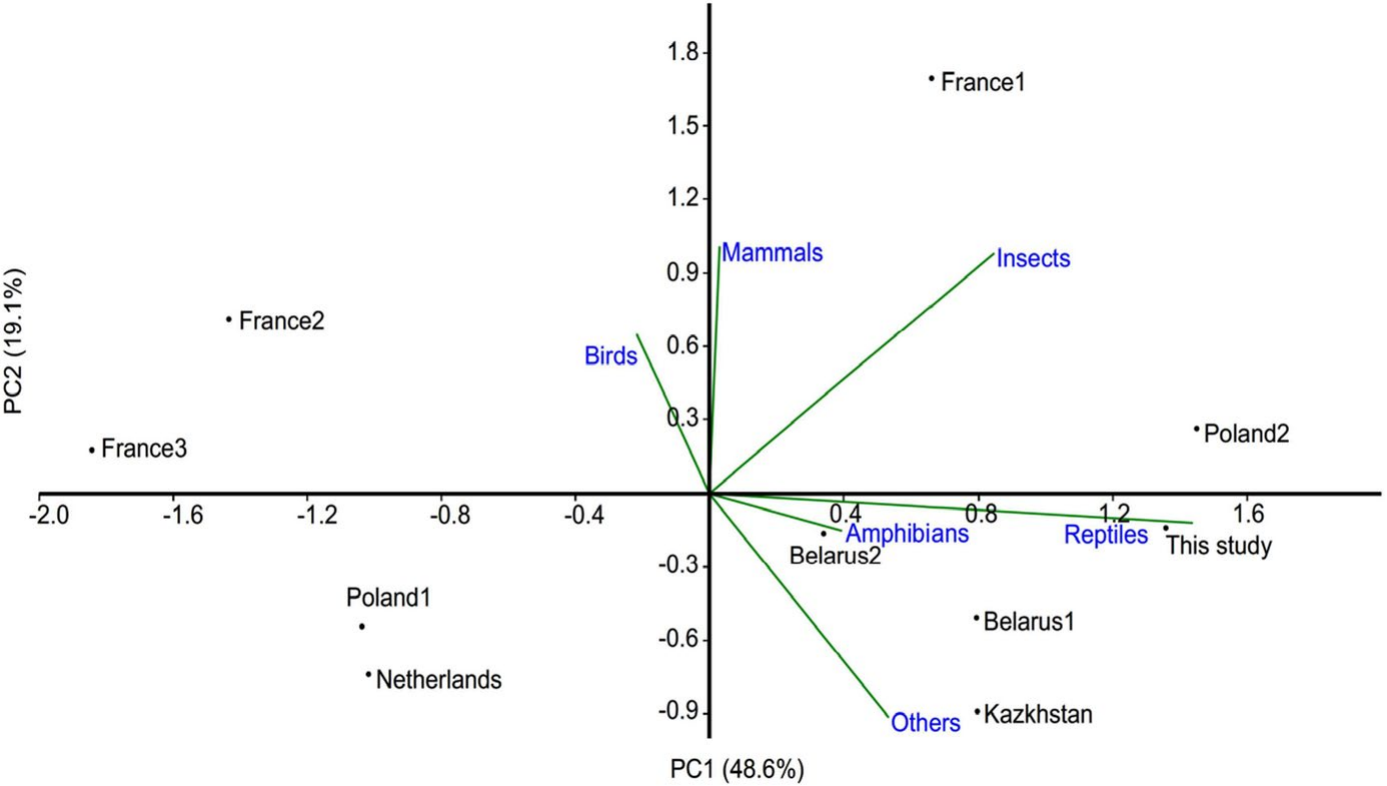
Principal component analysis biplot of diet composition of Montagu's Harriers in various breeding sites based on log(x + 1)‐transformed data on abundance of particular prey categories (reconstructed based on pellet composition). Sources of data name (site, region, main hunting habitat): Belarus1 (Belarus, Grodno region, farmland)—Vintchevski, 2009, Belarus2 (Belarus, Grodno region, farmland)—(Vintchevski & Yasievitch, 2009), France1 (Champagne, Ardenne Region, Barrois1, farmland)—(Millon et al., 2002), France2 (Champagne, Ardenne Region, Barrois2, farmland)—(Millon et al., 2002), France3 (Champagne–Ardenne Region, Crayeuse, farmland)—(Millon et al., 2002), Kazakhstan (Naurzum, Turgai Plateau, forest‐steppe transition zone)—(Terraube et al., 2010), The Netherlands (Groningen Province, farmland)—(Koks et al., 2002), Poland1 (Podlasie and Masovia regions, farmland)—(Mirski et al., 2009), Poland2 (Poland, Podlasie, farmland)—(Mirski et al., 2016), This study (Poland, Lublin region, farmland)

Principal component analysis performed on data on diet composition in various populations revealed that the first axis (explaining 48.6% of total variance) was the most correlated with reptiles (*r* = 0.79) while the second axis (explaining 19.1% of total variability) with mammals (*r* = 0.56) and insects (*r* = 0.54) (Figure 8).

Our summary of the Montagu's Harrier diet composition (Figure 8; Table 6) indicates that diet of the Chełm calcareous peat bogs population is the most similar to diet of birds from closely located site in Poland2 (Podlasie) (Figure 8). In both areas, insects constituted the most important part of prey abundance (> 71%) and reptiles were significantly represented. In all location in Poland1 and France1, insects constituted considerable part of the diet (>70% of abundance); in all other sites, their contribution was negligible (<14%). Reptiles serve as an important prey item (54%) only in the study from Kazakhstan, Netherlands, and France2 (>30%); in other sites, their contribution to the total prey abundance was negligible (< 20%). Contribution of mammals into diet of the Chełm calcareous peat bog population was considerably smaller than in majority of other sites except (>45%) from Poland and Kazakhstan with similar or even lower share of this prey item (10%–17%) (Table 6).

**TABLE 6:** Summary of Montagu's Harrier main prey item abundance (%) in various areas

Sampling site	Amphibians	Reptiles	Birds	Mammals	Insects	Others
Poland1	0.00	0.00	12.06	12.70	**75.24**	0.00
Poland2	0.02	2.34	6.57	9.94	**80.60**	0.53
Kazakhstan	0.00	54.19	14.34	17.13	13.59	0.74
France1	0.00	0.50	7.00	49.90	42.59	0.00
France2	0.00	0.00	31.41	**65.08**	3.51	0.00
France3	0.00	0.00	**53.48**	45.91	0.60	0.00
The Netherlands	0.00	0.00	35.91	**51.52**	7.18	5.39
Belarus1	0.12	5.16	18.94	**67.04**	5.81	2.93
Belarus2	0.09	0.92	16.96	**73.95**	6.60	1.49
This study	1.61	5.84	4.23	17.16	**70.80**	0.36

Note

All studies based on pellet analyses. Values > 50% bolded. Data sources and location: see Figure 10 caption.

## 4 DISCUSSION

### 4.1 Factors affecting diet composition in the studied population

Our study revealed that the Montagu's Harrier pellet composition (both regarding abundance and biomass) in 2007 was different than in 2008 and 2009 (Figure 2). Those differences may be linked to interannual differences in air temperatures noted in the vicinity of the studied area. 2007 was significantly warmer than 2009; the year 2008 was characterized by intermediate temperatures not significantly different from other studied seasons (Figure 7).

In the warmest 2007, we found the highest abundance of other insect and amphibian prey categories and surprisingly the lowest abundance of thermophilic reptiles and Field Crickets. Those results may be explained in terms of some factors affecting the abundance and availability of particular prey types or by prey switching, that is, focusing of Montagu's Harriers on other abundant, easy to catch prey. The Field Cricket is known to have high winter mortality of its larval stage (nymphs) (Hochkirch et al., 2006; Remmert, 1992). Thus, it cannot be excluded that mortality of nymphs in winter 2006/2007 was high which resulted in low availability of crickets for the studied population of Montagu's Harriers in summer 2007.

In contrast to our expectations, we did not detect within‐breeding season trends in number of ectothermic prey (insects and reptiles) or characterized by seasonal peak of availability (birds). This result may be affected by high availability of an alternative prey providing necessary energy content and nutritional value and/or lack of significant correlation between thermophilic prey and meteorological conditions, especially air temperatures.

### 4.2 Temporal variation in the Montagu's Harrier diet

We found some changes in diet composition of the studied population compared to studies from the 1990s. They included increase of abundance of insects and reptiles, decrease in number of mammal (especially voles) and avian prey, and presence of new prey species. We interpret increasing role of thermophilic prey (insects and reptiles) in context of ongoing climate changes and changes in foraging habitats. Recent studies indicate higher interannual survival of thermophilic organisms including insects and reptiles induced by higher winter temperatures (e.g., Bale & Hayward, 2010; Urban et al., 2014).

Decrease in abundance of small mammals in a diet and presence of new prey species may be interpreted in the context of agriculture‐induced changes in foraging habitats. The Montagu's Harrier and other harriers from *Circus* genus rely mainly on acoustic cues when detecting prey they prefer to forage in open grasslands, with low and barren vegetation (hay meadows and mow pastures in the studied peat bogs) enabling effective prey detection during cruising flights (dominating prey searching technique) (Bildstein, 2017; Clarke, 1996; Rice, 1982). After intensive changes in Polish agriculture after 1990s, main foraging grounds of the studied population of Montagu's Harriers (located in farmlands outside the calcareous peat bogs) were subject to strong transformations including increase in area of habitats covered with high vegetation as a result of wide scale abandonment of herbaceous vegetation mowing and grazing, land fallowing, and strong expansion of rapeseed and maize cultivation (Buczek & Buczek, 2017; Wojtak & Kitowski, 2003). Those transformations resulted from considerable decrease in the number of cows and sheep in E Poland (including the studied area) recorded in early 1990s (Dzun, 2012; Ptasinska‐Marcinkiewicz, 2014) and continuing until the years of the study (Główny Urząd Statystyczny, 2020). After the accession of Poland to the European Union in 2004, rapidly some new processes have been observed in the studied area. They include disappearance of livestock on meadows and pastures, homogenization of land use through aggregation of smaller fields. This is accompanied by the disappearance of various microhabitats increasing biodiversity as woodlots, boundary strips, or small water bodies (Kitowski I.—unpublished data). All mentioned changes in foraging habitats forced the studied population to forage on new prey items in traditional foraging areas or/and to explore a new, often suboptimal foraging grounds. Presence of novel prey items including synanthropic mammals like Brown Rat or House Mice indicates hunting and breeding very close to the buildings in consequences of decreasing anthropophobia documented in the Montagu's Harrier in E Poland (Pagorski & Krupinski, 2016). The presence of some new species not reported in 1985–1989 (Krogulec, 1992) as European Hamsters or European Water Vole may be explained by more frequent exploitation of habitats not used in the past.

Observed processes including decrease in contribution of mammals, especially voles many have some demographic consequences for the local population. Raptors, both diurnal and nocturnal including the Montagu's Harrier, are classified as income breeders gaining nutrients for egg production locally during the prelaying period (Durant et al., 2000; Stephens et al., 2009). Indeed, Montagu's Harriers just arrived from the wintering quarters forage intensively (Krogulec, 1992). One may expect that quality and quantity of food ingested during this period will affect considerably egg and offspring quality. Thus, significant decrease in the number of consumed voles may have affected negatively reproductive performance of the studied population. *Microtus* vole is considered as a high‐energy prey (energy density of one individual is estimated at 52.8 kJ (Masman et al., 1986)) and important source of calcium and other elements (Fritsch et al., 2010; Jankovska et al., 2009; Sawicka‐Kapusta et al., 1990). It has been found in Spanish population of the Montagu's Harrier that the proportion of mammalian prey, that is, rabbits in the prelaying diet was significantly related to breeding performance, with higher nest success and productivity in years when diet was dominated by hares (Arroyo & Garcia, 2006). Thus, it cannot be excluded that deterioration of reproductive performance (significant decrease of mean egg volume, mean clutch volume per female, mean dimension of eggs, and number chicks hatched) and increased (up to 75%) brood losses (Wiacek, 2015) leading to total disappearance of the breeding population in 2016 (Buczek & Buczek, 2017) were at least partially affected by changes in diet.

### 4.3 Comparison to other breeding populations

As we expected, we found that the diet of Montagu's Harrier breeding in the Chełm calcareous peat bog population is the most similar to diet of birds from closely located site in Poland2 (Podlasie, NE Poland). In both areas, insects constituted the most important part of prey abundance (> 71%) and reptiles were significantly represented. The diet of the crop‐breeding population from Poland2 during the chick‐rearing period consisted mainly of grasshoppers, beetles, Common Voles *Microtus arvalis*, Skylarks *Alauda arvensis,* and Yellow Wagtails *Motacilla flava* (Mirski et al., 2009). In Poland2 population, grasshoppers during the chick‐rearing period made up 54% of abundance and 8% of biomass (Mirski et al., 2009) while we did not find this type of prey at this stage of breeding. However, we recorded grasshoppers in other stages of breeding but always with relatively low contribution to the total abundance (<5.0%) (Table 6). This type of prey plays very important role in development of hunting skills in juvenile Montagu's Harriers. Adults use this type of prey as prey model during an active training of fledglings (Kitowski, 2005). Parental birds encourage fledglings to leave the fens and fly to neighboring arable lands abundant in grasshoppers. This prey item was the first one self‐hunted by the juveniles (Kitowski, 2005).

### 4.4 Limitations of the study

We are aware of some limitations of our study. We based our study on pellets produced by unknown number of individuals. Comparison of various techniques to study diet of the Hen Harrier (*Circus cyaneus*) revealed that pellets over‐represent mammalian prey and under‐represent avian prey; however, they are useful for estimating prey diversity and the frequency of certain prey types in the diet (Redpath et al., 2001). Data presented here, however, base on method not disturbing birds on the nest what is especially important in the case of declining population. Moreover, existence of historical data on diet of Montagu's Harriers from the studied population (based on the same technique—pellet analysis) gave us an opportunity to study temporal changes in abundance of the most important prey categories. Our study has filled an evident gap in knowledge about within‐ and between‐season diet variation in the Montagu's Harrier.

## 5 CONCLUSIONS

We found that the diet of Montagu's Harrier breeding in calcareous peat bogs (Chełm, Eastern Poland) and foraging in nearby agricultural landscape is dominated by insects and mammals (by number) and mammals and birds (by biomass). Biomass and abundance of main prey items differed between the years characterized by different air temperatures. Comparison with historical data collected in the same areas revealed that currently diet of the studied population is richer in thermophilic prey items, such as insects and reptilians, and poorer in small mammals and birds. This may be linked to climate change‐driven changes in prey composition in foraging grounds around the calcareous marshes. Our comparison with other studies revealed that the diet of the Montagu's Harrier population breeding in the calcareous marshes near Chełm is the most similar to the geographically closest populations foraging in similar habitats. Diet of all those populations is characterized by high contribution of insects. Our study revealed that the Montagu's Harrier population breeding in calcareous peat bogs in SE Poland was able to respond to changes in foraging habitats and prey composition by opportunistic foraging on easily available prey. However, they probably were not able to fully compensate lower contribution of important diet components (e.g., energy‐rich voles) which may have resulted in deterioration of reproductive performance and finally in total collapse of the studied population in 2010s.

## CONFLICT OF INTEREST

The authors declare no conflicts of interest.

## AUTHOR CONTRIBUTIONS

Ignacy Kitowski: Conceptualization (Lead); Data curation (Lead); Investigation (Lead); Resources (Lead); Writing‐original draft (Lead); Writing‐review & editing (Lead). Dariusz Jakubas: Conceptualization (Supporting); Formal analysis (Lead); Methodology (Lead); Visualization (Lead); Writing‐original draft (Lead); Writing‐review & editing (Lead). Paweł Mirski: Investigation (Supporting); Writing‐review & editing (Supporting). Grzegorz Pitucha: Investigation (Supporting); Writing‐review & editing (Supporting). Kornelia Markowska: Investigation (Supporting); Writing‐review & editing (Supporting).

## Data Availability

Data of the Montagu's Harrier diet are available at Dryad: https://doi.org/10.5061/dryad.kkwh70s42
